# Prediction of disease-related genes based on weighted tissue-specific networks by using DNA methylation

**DOI:** 10.1186/1755-8794-7-S2-S4

**Published:** 2014-10-22

**Authors:** Min Li, Jiayi Zhang, Qing Liu, Jianxin Wang, Fang-Xiang Wu

**Affiliations:** 1 School of Information Science and Engineering, Central South University, Changsha 410083, Hunan, P. R. China; 2 College of Engineering, University of Saskatchewan, 57 Campus Dr., Saskatoon, SK Canada

## Abstract

**Background:**

Predicting disease-related genes is one of the most important tasks in bioinformatics and systems biology. With the advances in high-throughput techniques, a large number of protein-protein interactions are available, which make it possible to identify disease-related genes at the network level. However, network-based identification of disease-related genes is still a challenge as the considerable false-positives are still existed in the current available protein interaction networks (PIN).

**Results:**

Considering the fact that the majority of genetic disorders tend to manifest only in a single or a few tissues, we constructed tissue-specific networks (TSN) by integrating PIN and tissue-specific data. We further weighed the constructed tissue-specific network (WTSN) by using DNA methylation as it plays an irreplaceable role in the development of complex diseases. A PageRank-based method was developed to identify disease-related genes from the constructed networks. To validate the effectiveness of the proposed method, we constructed PIN, weighted PIN (WPIN), TSN, WTSN for colon cancer and leukemia, respectively. The experimental results on colon cancer and leukemia show that the combination of tissue-specific data and DNA methylation can help to identify disease-related genes more accurately. Moreover, the PageRank-based method was effective to predict disease-related genes on the case studies of colon cancer and leukemia.

**Conclusions:**

Tissue-specific data and DNA methylation are two important factors to the study of human diseases. The same method implemented on the WTSN can achieve better results compared to those being implemented on original PIN, WPIN, or TSN. The PageRank-based method outperforms degree centrality-based method for identifying disease-related genes from WTSN.

## Background

With the completion of HGP (Human Genome Project) and the development of high-throughput technologies, more and more protein-protein interaction data can be obtained, which makes it possible for us to study the life activity at the network level [1-3]. Many network-based methods have been proposed to predict protein functions, identify essential proteins and disease-related genes and complexes [5-7]. It has been shown that the network-based disease-related gene discovery approaches can achieve comparable qualities with current integrative methods [8-11]. More and more attentions have been paid to discover disease-related genes by using network-based methods. Moti et al. [12] developed a neighbourhood-based algorithm to predict disease genes using protein-protein interactions by using the associated intervals. In a similar way, Kar et al. [13], Chavali et al. [14], and Sun et al. [15] further analyzed different diseases and demonstrated that the topological features of genes in associated intervals were different in the corresponding networks. Xu et al. [16] predicted disease-related genes by using topological features to improve KNN clustering algorithm. Sun et al. [17] used clustering analysis method to predict human disease-related gene clusters based on the network. In addition, some typical graph partitioning methods and clustering approaches, such as GS[18], MCL [19], VI-Cut [20], IPCA [21], MSCF [22], HC-PIN[23], RW[24], and their improved algorithms, can also be used to discover candidate disease-related genes.

Although great progresses have been made on the network-based methods, it is still a challenge task to identify disease-related genes as the considerable false-positives are still existed in the current available PINs [25]. To reduce the effect of false-positives, researchers started to integrate different types of biological information, such as gene expression profiles [26-29], orthology data [30], gene ontology annotations [31], and DNA methylation [32], into protein interaction networks. Accumulated studies suggested that DNA methylation may cause changes of chromatin structure, DNA conformation, DNA stability, and interaction mode between DNA and proteins, and such aberrant conditions may cause cancers[32]. Therefore, DNA methylation information can be used to improve identification of disease-related genes. For prioritizing cancer-related genes, Liu et al. [32] constructed a weighted human protein interaction network by using DNA methylation correlations.

Recently, some researchers tried to develop new network-based methods for finding disease-related genes by using tissue-specific networks (TSN). Tissue specificity is an important aspect of many genetic diseases and the majority of genetic disorders tend to manifest only in a single or a few tissues [33-35]. Magger et al. [36] believed that the predicted precision would be influenced when the same data sets were used to predict disease-related genes of different tissues and diseases. Tissue-specific networks can reflect the features of related tissues of diseases better, and usage of the network will enhance the accuracy of predicting disease-related genes.

In this paper, we constructed a weighted tissue-specific network (WTSN) by integrating human protein interaction network, DNA methylation, and tissue-specific data. A PageRank-based method was developed to identify disease-related genes from the constructed WTSN. To validate the effectiveness of the constructed network and the proposed method, we tested them on the prediction of disease-related genes of colon cancer and leukemia. The experimental results show that tissue-specific data and DNA methylation are two important factors to the study of human disease. The same method implemented on the WTSN can achieve better results compared to those being implemented on original PIN, WPIN, or TSN. The PageRank-based method outperforms degree centrality-based method for identifying disease-related genes from WTSN.

## Methods

### Materials

The human protein-protein interactions were downloaded from DIP [37], IntAct [38], MINT [39], BioGRID [40], HPRD [41], Uniprotkb and HGNC databases. These protein-protein interactions were combined to construct a PIN by filtering the self-interactions and repeated interactions. The final PIN comprises 15,389 human proteins and 108,317 physical interactions.

To determine tissue-specific interactions, gene expression microarray data GSE1133 and gene identity matched data GPL96 [42] were used to extract expression values of each gene in different tissues. All the co-expression relationships in 79 tissues were marked by binary variables. 79 tissues were mathematically represented in a matrix of 108,317 interactions.

DNA methylation information was downloaded from GSE17648 [43] and GSE28462 data sets of GEO. Aberrant methylation information was downloaded from PubMeth [44] database. Gene signing messages of related diseases in the gene signatures bank were downloaded from GeneSigDB [45].

### Construction of WTSN

**Figure 1:**
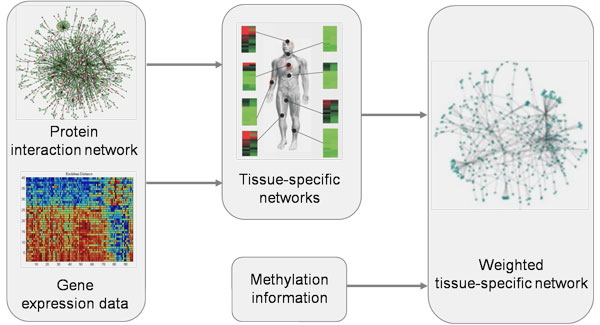
**Steps of constructing WTSN**.

As shown in Figure 1, we constructed the WTSNs according to the following three steps:

Step 1: Construct the human PIN by integrating protein-protein interaction data obtained from DIP [37], IntAct [38], MINT [39], BioGRID [40], HPRD [41], Uniprotkb and HGNC databases and filtering the self-interactions and repeated interactions.

Step 2: For a special disease, construct its TSN by using the PIN, tissue-specific data and disease-tissue association data.

Step 3: Weight the constructed TSN by using DNA methylation data.

In step 2, to construct the TSN for a special disease, we first obtained the relationship between diseases and tissues from the works of Lage et al [46]. From the disease-tissue association data we can extract the tissues which are related to the special disease. Tissue-specific subnetworks were constructed by using gene expression microarray data GSE1133 and GPL96. When constructing the tissue-specific subnetworks we also used the mode removal method which has been used by Waldman et al. [34], Bossi et al. [35] and Lopes et al. [47]. The main idea of the mode removal method is to construct tissue-specific subnetworks through removing unexpressed genes in related tissues. There are 79 different human cells or gene expression data of tissues. For each gene, its normalized expression level is calculated and it is considered to be expressed in the tissue [33] if its normalized expression level is larger than a certain threshold. For each tissue, a corresponding subnetwork is generated by removing the unexpressed nodes and its interactions from the PIN. Then, a final TSN can be constructed by combing the subnetworks for all the disease-related tissues.

In step 3, we further weighted the TSN by using DNA methylation data. For two connected proteins in TSN, the Pearson Correlation Coefficient (PCC) [48-50], as shown in formula (1), was calculated to assess the two proteins' association of the methylation.

(1) $$PCC\left(X,Y\right)=\frac{\sum x_{i}\cdot y_{i}\frac{\sum x_{i}\cdot \sum y_{i}}{N}}{\sqrt{\left(\sum x_{i}^{2}\frac{{\left(\sum x_{i}\right)}^{2}}{N}\right)\cdot \left(\sum y_{i}^{2}\frac{{\left(\sum y_{i}\right)}^{2}}{N}\right)}}$$

where X and Y are two proteins which interact with each other in TSN. The variables

*x_i _*and *y_i _*denote the corresponding DNA methylation value of protein X and protein Y at i^th ^point, respectively. N indicates the total number of methylation data for each protein in TSN.

### PageRank-based method

The PageRank algorithm was first proposed by Brin and Page [51], which was used to evaluate webpage and produce an authority value to show the importance of each webpage. The main idea of PageRank is to suppose that a random walker selects chains to be visited according to uniform probability distribution. As the PIN is generally considered as an undirected graph, we implemented PageRank on undirected graph in this paper. It has been shown that the PageRank of an undirected graph is statistically close to the degree distribution. Hence, we compared the results of PageRank-based method and degree-centrality-based method in the section of results. The PageRank algorithm was computed in an iterative way where the probability distribution is used as the input of the next walking of this process.

In this paper, we treated known aberrant methylation genes as seed nodes and set initial quantity value with the use of the seed set, which will enhance the importance of seed nodes in network and solve defects of initial PageRank algorithm. The aberrant methylation data related to specific diseases in PubMeth database [44] were used in this paper.

Before using the PageRank-based method to predict disease-related genes, we perturbed the DNA methylation data 1000 times and recalculated the PCC of the random DNA methylation for the gene pairs in each perturbed methylation dataset. Then, 1000 random WTSNs were obtained and the PageRank algorithm was applied on these 1000 random WTSNs. The average PageRank value for each protein was calculated on the 1000 random WTSNs and it is considered to be distinctiveness if its PageRank value on the WTSN is higher than the average PageRank value on the 1000 random WTSNs. All the proteins in the WTSN were ranked according to their PageRank values in original WTSN and those distinctiveness proteins will be outputted as candidate disease-related genes.

### Results and discussion

To validate the effectiveness of the constructed WTSN and the PageRank-based method, we used four different types of PINs: original human PIN, TSN for each special disease, WPIN by using DNA methylation, and WTSN. We applied the PageRank-based method to these four different types of protein interaction networks, and the corresponding results were marked as PR, SPR, WPR, SWPR, respectively. Two important cancers of colon cancer and leukemia were used as case studies here. For each test, the predicted precision was calculated by using the following formula:

(2) $$precision=M_{Signature}/N_{Prediction}$$

where *N_Prediction _*is the number of predicted candidate disease-related genes and *M_Signature _*is the number of disease-related genes which can be found in GeneSigDB [52] with the corresponding signatures.

In this paper, the improved PageRank algorithm by using significant analysis is also compared to the degree centrality-based method. In 2011, Liu et al. [32] identified potential disease-related genes by using weighted degree centrality based on the integration of DNA methylation and protein-protein interaction data. Degree centrality (DC) of a given protein in an unweighted network is defined as the number of nodes that directly connect to it. The weighted degree centrality (WDC) of a given protein in a weighted network is defined as the sum of weights of edges connecting the given node and its neighbors. We marked the results of DC as SDC and SWDC when it being applied on the TSN and the WTSN, respectively.

### Identification of disease-related genes from TSN

**Figure 2:**
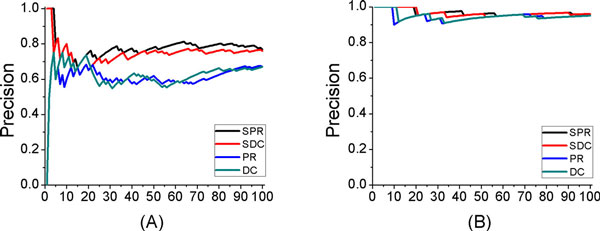
**Comparison of results for PageRank and degree centrality when being applied on the original protein interaction network (PIN) and tissue specific network (TSN)**. The x axis represents the number of identified disease-related genes. The y axis represents the predicted precision of each result. (A) Results on colon cancer. (B) Results on leukemia.

To analyze whether the tissue specific information is useful to the identification of disease-related genes, we first applied the PageRank-based method by using significant analysis and degree centrality-based method to the original PIN and the TSN. The experimental results on colon cancer and leukemia were shown in Figure 2. From Figure 2 we can see that both the PageRank method and degree-centrality-based method achieved better results when being applied on TSN than being applied on PIN for colon cancer and leukemia. Especially for colon cancer, the predicted precision of the PageRank algorithm and degree-centrality were improved more than 20% on average when predicting no more than 100 candidate disease-related genes. For the same cancer, the PageRank-based method by using significant analysis performs a little better than degree centrality-based method when being applied on TSN.

### Identification of disease-related genes from WTSN

**Figure 3:**
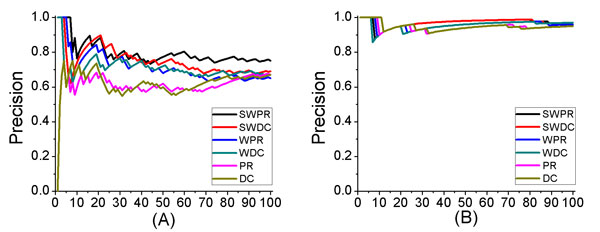
**Comparison of results for PageRank and degree centrality when being applied on the original protein interaction network (PIN), weighted protein interaction network (WPIN), and weighted tissue specific network (WTSN)**. The x axis represents the number of identified disease-related genes. The y axis represents the predicted precision of each result. (A) Results on colon cancer. (B) Results on leukemia.

In the above subsection, we have shown that tissue specific information contributes to the accurate identification of disease-related genes. In this subsection, we further evaluated the effectiveness of weighting by using DNA methylation and compared the results on WPIN and PIN. As shown in Figure 3 (A), both the PageRank-based method and the degree-centrality-based method achieved better results when being applied on WPIN than on PIN for predicting no more than 80 colon cancer-related candidate genes. Similar results were obtained for leukemia, as shown in Figure 3(B). However, the improvement is not so clear for the high predicted precision on leukemia. The improvement of predicted precision on the WPIN shows that weighting protein interaction network by using DNA methylation contributes to more accurate prediction of disease-related genes.

To analyze the contributions of combination of tissue-specific and DNA methylation, we showed the results of PageRank-based method and Degree-centrality-based method both on WPIN and WTSN. From Figure 3 we can see that the predicted precisions of PageRank-based method and Degree-centrality-based method were both further improved when being applied on WTSN. As shown in Figure 3(A), the precision of SWPR is 1.2 times more than that of WPR for predicting no more than 100 colon cancer-related candidates. On the WTSN, the PageRank-based method still performs a little better than the degree centrality-based method. Hence, we further analyzed the candidate disease-related genes predicted by the PageRank-based method. Table 1 and Table 2 showed the top 20 candidate disease-related genes of colon cancer and Leukemia, respectively, which were identified by the PageRank method with significant analysis from PIN, TSN, WPIN, WTSN.

**Table 1:** Top 20 candidate disease-related genes identified by PageRank method with significant analysis from PIN, TSN, WPIN, WTSN, for colon cancer, respectively.

WTSN		WPIN		TSN		PIN	
**Genes**	**In GeneSigDB**	**Genes**	**In GeneSigDB**	**Genes**	**In GeneSigDB**	**Genes**	**In GeneSigDB**

CDH1	**yes**	APC	**yes**	NR3C1	**yes**	UBC	no

FAS	**yes**	CDH1	**yes**	MLH1	**yes**	ELAVL1	**yes**

CD44	**yes**	FAS	**yes**	CDH1	**yes**	SUMO2	**yes**

THBS1	**yes**	CD44	**yes**	CDKN2A	**yes**	MYC	**yes**

TIMP3	**yes**	THBS1	**yes**	ATM	no	GRB2	no

GSTP1	**yes**	STK11	no	FAS	**yes**	SUMO1	**yes**

CREBBP	**yes**	GSTP1	**yes**	MGMT	no	SNCA	no

STK11	no	UBC	no	THBS1	**yes**	ESR1	**yes**

HDAC1	**yes**	ALX4	no	CD44	**yes**	GABARAPL2	no

UBC	no	HRK	**yes**	RASSF1	**yes**	TP53	**yes**

NCL	**yes**	TIMP3	**yes**	STK11	no	YWHAZ	**yes**

CCND1	**yes**	SRC	**yes**	DAPK1	**yes**	GABARAPL1	**yes**

PRKDC	**yes**	NCL	**yes**	CHFR	no	GABARAP	no

SFN	**yes**	CREBBP	**yes**	GSTP1	**yes**	TRAF6	**yes**

ELAVL1	**yes**	HDAC1	**yes**	UBC	no	RAD23A	**yes**

TGM2	**yes**	SFRP1	**yes**	TIMP3	**yes**	EP300	no

FYN	no	ELAVL1	**yes**	HSP90AA1	**yes**	EGFR	**yes**

FN1	**yes**	CCND1	**yes**	TP53	**yes**	SRC	**yes**

ACTR3	**yes**	PRKDC	**yes**	SFRP1	**yes**	YWHAG	**yes**

MMP14	**yes**	FYN	no	PML	**yes**	ESR2	no

**Table 2:** Top 20 candidate disease-related genes identified by PageRank method with significant analysis from PIN, TSN, WPIN, WTSN, for Leukemia, respectively.

WTSN		WPIN		TSN		PIN	
**Genes**	**In GeneSigDB**	**Genes**	**In GeneSigDB**	**Genes**	**In GeneSigDB**	**Genes**	**In GeneSigDB**

ABL1	**yes**	ESR1	**yes**	ESR1	**yes**	ESR1	**yes**

CDKN1A	**yes**	ABL1	**yes**	ABL1	**yes**	ABL1	**yes**

MLH1	**yes**	CDKN1A	**yes**	MLH1	**yes**	RB1	**yes**

MGMT	**yes**	CCND1	**yes**	RB1	**yes**	CDKN1A	**yes**

LMNA	**yes**	CDKN2A	**yes**	CDKN1A	**yes**	RARA	**yes**

NR0B2	**yes**	MLH1	**yes**	CDKN2A	**yes**	MLH1	**yes**

CHFR	**yes**	PARK2	no	PTPN6	**yes**	CDH1	**yes**

DIABLO	**yes**	MME	**yes**	SYK	**yes**	PTPN6	**yes**

CEBPD	**yes**	MYOD1	**yes**	PTEN	**yes**	CDKN2A	**yes**

DAPK1	**yes**	LMNA	**yes**	MGMT	**yes**	PARK2	no

GRB2	**yes**	NR0B2	**yes**	HCK	**yes**	SYK	**yes**

ACTB	**yes**	MGMT	**yes**	THBS1	**yes**	CCND1	**yes**

HDAC1	**yes**	APAF1	**yes**	LMNA	**yes**	TP73	**yes**

HSP90AA1	**yes**	PGR	**yes**	NR0B2	**yes**	MYOD1	**yes**

PAX6	**yes**	AHR	**yes**	UBC	**yes**	PTEN	**yes**

FYN	**yes**	DAPK1	**yes**	CHFR	**yes**	LMNA	**yes**

EEF1A1	**yes**	PAX6	**yes**	DAPK1	**yes**	THBS1	**yes**

JAK1	**yes**	CIITA	**yes**	GSTP1	**yes**	MGMT	**yes**

CRKL	**yes**	CHFR	**yes**	RARB	**yes**	HCK	**yes**

CCNB1	**yes**	HIC1	**yes**	DIABLO	no	NR0B2	**yes**

As shown in Table 1, out of the top 20 candidate disease-related genes of colon cancer, 17 genes were found to have gene signatures in GeneSigDB. The number of true colon cancer-related genes identified from WTSN is higher than that identified from PIN, WPIN and TSN. For the unknown gene STK11 identified from WTSN, we found that it has been reported to contribute to the development of both sporadic and familial forms of cancer and germline and somatic genetic alterations of the STK11/LKB1 gene may play a causal role in carcinogenesis [53]. For leukemia, we were delighted to see that all the top 20 candidate disease-related genes were included in GeneSigDB. The top 100 candidate disease-related genes of colon cancer and Leukemia can be seen in additional file 1 and additional file 2 respectively.

## Conclusion

The primary purpose of this study is to use WTSN to predict disease-related genes. We proposed a PageRank-based method to identify disease-related genes from WTSN. Firstly, TSN was constructed by combining PIN and gene expression data. Secondly, WTSN was constructed by weighting TSN with methylation information. Finally, an improved PageRank algorithm was used to predict disease-related genes by using significance analysis based on WTSN. To validate the effectiveness of the proposed method, we constructed PIN, WPIN, TSN, WTSN for colon cancer and leukemia, respectively. The experimental results on colon cancer and leukemia show that the combination of tissue-specific data and DNA methylation can help to identify disease-related genes more accurately. Moreover, the PageRank-based method was effective to predict disease-related genes on the case studies of colon cancer and leukemia.

## Competing interests

The authors declare that they have no competing interests.

## Authors' contributions

JYZ and QL obtained the protein-protein interaction data, tissue-specific data, methylation information and GeneSigDB. ML, JYZ and QL designed the method to predict disease-related genes. JYZ and ML analyzed the results. ML, JXW and FXW drafted the manuscript together. All authors have read and approved the manuscript.

## Supplementary Material

Additional File 1 The top 100 candidate disease-related genes of colon cancer Click here for file

Additional File 2 The top 100 candidate disease-related genes of Leukemia Click here for file

## Declarations

The publication costs for this article were funded by the National Natural Science Foundation of China under Grant No. 61370024.

This article has been published as part of *BMC Medical Genomics *Volume 7 Supplement 2, 2014: IEEE International Conference on Bioinformatics and Biomedicine (BIBM 2013): Bioinformatics in Medical Genomics. The full contents of the supplement are available online at http://www.biomedcentral.com/bmcmedgenomics/supplements/7/S2.

## References

[B1] Wang J Li M Deng Y Pan Y Recent advances in clustering methods for protein interaction networks BMC Genomics 2010 11 Suppl 3 S10 10.1186/1471-2164-11-S3-S10 21143777 PMC2999340

[B2] Li M Wu X Wang J Pan Y Towards the identification of protein complexes and functional modules by integrating PPI network and gene expression data BMC Bioinformatics 2012 13 1 109 10.1186/1471-2105-13-109 22621308 PMC3434013

[B3] Zhao B Wang J Li M Wu F-X Pan Yi Detecting Protein Complexes Based on Uncertain Graph Model IEEE/ACM Transactions on Computational Biology and Bioinformatics 2014 11 3 486 497 10.1109/TCBB.2013.2297915 26356017

[B4] Keong H Mason SP Barabai AL Lethality and centrality in protein networks Nature 2001 411 6833 41 42 10.1038/35075138 11333967

[B5] Zhong J Wang J Peng W Prediction of essential proteins based on gene expression programming BMC Genomics 2013 14 4 18 24267033 10.1186/1471-2164-14-S4-S7 PMC3856491

[B6] Wang J Peng W Wu F X Computational approaches to predicting essential proteins: a survey PROTEOMICS-Clinical Applications 2013 7 1-2 181 192 10.1002/prca.201200068 23165920

[B7] Wang J Li M Wang H Identification of essential proteins based on edge clustering coefficient Computational Biology and Bioinformatics 2012 9 4 1070 1080 IEEE/ACM Transactions on 22084147 10.1109/TCBB.2011.147

[B8] Saket Navlakha Carl Kingsford The power of protein interaction networks for associating genes with diseases Bioinformatics 2010 26 8 1057 1063 10.1093/bioinformatics/btq076 20185403 PMC2853684

[B9] Lage K A human phenome-interactome network of protein complexes implicated in genetic disorders Nature Biotechnol 2007 25 23 309 316 17344885 10.1038/nbt1295

[B10] Wu X Network-based global inference of human disease genes Molecular Systems Biology 2008 4 1 189 18463613 10.1038/msb.2008.27 PMC2424293

[B11] Linghu B Genome-wide prioritization of disease genes and identification of disease-disease associations from an integrated human functional linkage network Genome Biology 2009 10 R91 10.1186/gb-2009-10-9-r91 19728866 PMC2768980

[B12] MOti Snel B Huynen MA Brunner HG Predicting disease genes using protein-protein interactions interactions Journal of medical genetics 2006 43 8 691 698 10.1136/jmg.2006.041376 16611749 PMC2564594

[B13] Gozde Kar Attila Gursoy Ozlem Keskin Human Cancer Protein-Protein Interaction Network: A Structural Perspective PLoS Computational Biology 2009 5 12 1 18 10.1371/journal.pcbi.1000601 PMC2785480 20011507

[B14] Sreenivas Chavali Fredrik Barrenas Kartiek Kanduri Network properties of human disease genes with pleiotropic effects BMC Systems Biology 2010 4 1 78 10.1186/1752-0509-4-78 20525321 PMC2892460

[B15] Jingchun Sun Peilin Jia Ayman Fanous H Schizophrenia Gene Networks and Pathways and Their Applications for Novel Candidate Gene Selection PLoS ONE 2010 5 6 e11351 10.1371/journal.pone.0011351 20613869 PMC2894047

[B16] Xianzhen Xu Yongjin Li Discovering disease-genes by topological features in human protein-protein interaction network Bioinformatics 2006 22 22 2800 2805 10.1093/bioinformatics/btl467 16954137

[B17] Sun PG Gao L Han S Prediction of human disease-related gene clusters by clustering analysis International journal of biological sciences 2011 7 1 61 21278917 10.7150/ijbs.7.61 PMC3030143

[B18] Shrivastava N Graph summarization with bounded error Proceedings of the 2008 ACM SIGMOD international conference on Management of data 2008 419 432 10.1145/1376616.1376661

[B19] Stijn Van Dongen Graph clustering via a discrete uncoupling process SIAM Journal on Matrix Analysis and Applications 2008 30 1 121 141 10.1137/040608635

[B20] Saket Navlakha Niranjan Nagarajan James White Navlakha S Rastogi R Finding Biologically Accurate Clusterings in Hierarchical Tree Decompositions Using the Variation of Information Journal of Computational Biology 2010 17 3 503 516 10.1089/cmb.2009.0173 20377460

[B21] Li M Chen J Wang J Modifying the DPClus algorithm for identifying protein complexes based on new topological structures BMC bioinformatics 2008 9 1 398 10.1186/1471-2105-9-398 18816408 PMC2570695

[B22] Ding X Wang W Peng X Wang J Mining protein complexes from PPI networks using the minimum vertex cut Tsinghua Science and Technology 2012 17 6 674 681 10.1109/TST.2012.6374369

[B23] Wang J Li M Chen J A fast hierarchical clustering algorithm for functional modules discovery in protein interaction networks IEEE/ACM Trans Comput Biol Bioinform 2011 8 3 607 620 10.1109/TCBB.2010.75 20733244

[B24] Erten Sinen Mehmet Koyutürk Role of Centrality in Network-Based Prioritization of Disease Genes[M]//Evolutionary Computation, Machine Learning and Data Mining in Bioinformatics Lecture Notes in Computer Science Evolutionary Computation, Machine Learning and Data Mining in Bioinformatics. Lecture Notes in Computer Science 2010 6023 13 25 10.1007/978-3-642-12211-8_2

[B25] Montanez G Cho Y-R Predicting False Positives of Protein-Protein Interaction Data by Semantic Similarity Measures Current Bioinformatics 2013 8 3 339 346 10.2174/1574893611308030009

[B26] Li M Zheng R Zhang H Wang J Pan Y Effective identification of essential proteins based on priori knowledge, network topology and gene expressions Methods 2014 67 3 325 333 10.1016/j.ymeth.2014.02.016 24565748

[B27] Wang J Peng X Peng W Dynamic protein interaction network construction and applications Proteomics 2014 8 4-5 338 352 24339054 10.1002/pmic.201300257

[B28] Wang J Peng X Li M Construction and application of dynamic protein interaction network based on time course gene expression data Proteomics 2013 13 2 301 312 10.1002/pmic.201200277 23225755

[B29] Tang X Feng Q Wang J Clustering based on multiple biological information: approach for predicting protein complexes IET systems biology 2013 7 5 223 230 10.1049/iet-syb.2012.0052 24067423 PMC8687320

[B30] Peng W Wang J Wang W Liu Q Wu FX Pan Y Iteration method for predicting essential proteins based on orthology and protein-protein interaction networks BMC systems biology 2012 6 1 87 10.1186/1752-0509-6-87 22808943 PMC3472210

[B31] Mahmoud Mahdavi A Yen-Han Lin False positive reduction in protein-protein interaction predictions using gene ontology annotations BMC Bioinformatics 2007 8 262 10.1186/1471-2105-8-262 17645798 PMC1941744

[B32] Liu H Su J Li J Prioritizing cancer-related genes with aberrant methylation based on a weighted protein-protein interaction network BMC systems biology 2011 5 1 158 10.1186/1752-0509-5-158 21985575 PMC3224234

[B33] Shlomi T Network-based prediction of human tissue-specific metabolism Nature Biotechnol 2008 26 9 1003 1010 10.1038/nbt.1487 18711341

[B34] Waldman YY Tuller T Shlomi T Translation efficiency in humans: tissue specificity global optimization and differences between developmental stages Nucleic Acids Research 2010 38 9 2964 2974 10.1093/nar/gkq009 20097653 PMC2875035

[B35] Bossi A Lehner B Tissue specificity and the human protein interaction network Molecular Systems Biology 2009 5 1 260 19357639 10.1038/msb.2009.17 PMC2683721

[B36] Magger O Waldman YY Ruppin E Enhancing the prioritization of disease-causing genes through tissue specific protein interaction networks PLoS Computational Biology 2012 8 9 e1002690 10.1371/journal.pcbi.1002690 23028288 PMC3459874

[B37] Xenarios I Rice DW Salwinski L DIP: the database of interacting proteins Nucleic Acids Res 2000 28 1 289 291 10.1093/nar/28.1.289 10592249 PMC102387

[B38] Kerrien S Alam-Faruque Y Aranda B Bancarz I Bridge A Derow C Dimmer E Feuermann M Friedrichsen A Huntley R IntAct-open source resource for molecular interaction data Nucleic acids research 2007 35 suppl 1 D561 D565 17145710 10.1093/nar/gkl958 PMC1751531

[B39] Ceol A Chatr Aryamontri A Licata L Peluso D Briganti L Perfetto L Castagnoli L Cesareni G MINT, the molecular interaction database: 2009 update Nucleic Acids Res 2010 38 suppl 1 D532 539 19897547 10.1093/nar/gkp983 PMC2808973

[B40] Stark C Breitkreutz BJ Reguly T BioGRID: a general repository for interaction datasets Nucleic acids research 2006 34 suppl 1 D535 D539 16381927 10.1093/nar/gkj109 PMC1347471

[B41] Keshava Prasad TS Goel R Kandasamy K Keerthikumar S Kumar S Mathivanan S Telikicherla D Raju R Shafreen B Venugopal A Human Protein Reference Database-2009 update Nucleic acids research 2009 37 suppl 1 D767 D772 18988627 10.1093/nar/gkn892 PMC2686490

[B42] Su AI Wiltshire T Batalov S A gene atlas of the mouse and human protein-encoding transcriptomes Proceedings of the National Academy Sciences of the United States of America 2004 101 16 6062 6067 10.1073/pnas.0400782101 PMC395923 15075390

[B43] Chen Yao Hongdong Li Xiaopei Shen Zheng He Lang He Zheng Guo Reproducibility and Concordance of Differential DNA Methylation and Gene Expression in Cancer PLoS ONE 2012 7 1 e29686 10.1371/journal.pone.0029686 22235325 PMC3250460

[B44] Ongenaert M Van Neste L De Meyer T PubMeth: a cancer methylation database combining text-mining and expert annotation Nucleic Acids Research 2008 36 suppl 1 D842 D846 17932060 10.1093/nar/gkm788 PMC2238841

[B45] Culhane AC Schwarzl T Sultana R GeneSigDB-a curated database of gene expression signatures Nucleic Acids Res 2012 40 D D1060 D1066 10.1093/nar/gkr901 19934259 PMC2808880

[B46] Lage K Hansen NT Karlberg EO A large-scale analysis of tissue-specific pathology and gene expression of human disease genes and complexes Proceedings of the National Academy of Sciences 2008 105 52 20870 20875 10.1073/pnas.0810772105 PMC2606902 19104045

[B47] Lopes TJ Schaefer M Shoemaker J Tissue-specific subnetworks and characteristics of publicly available human protein interaction databases Bioinformatics 2011 27 17 2414 2421 10.1093/bioinformatics/btr414 21798963

[B48] Tang X Wang J Zhong J Pan Y Predicting Essential proteins based on Weighted Degree Centrality Computational Biology and Bioinformatics, IEEE/ACM Transactions 2014 11 2 407 418 10.1109/TCBB.2013.2295318 26355787

[B49] Li M Zhang H Wang J Pan Y A new essential protein discovery method based on the integration of protein-protein interaction and gene expression data BMC systems biology 2012 6 1 15 10.1186/1752-0509-6-15 22405054 PMC3325894

[B50] Eggers JJ Bauml R Tzschoppe R Scalar costa scheme for information embedding Signal Processing, IEEE Transactions on 2003 51 4 1003 1019 10.1109/TSP.2003.809366

[B51] Brin S Page L The anatomy of a large-scale hypertextual Web search engine Computer networks and ISDN systems 1998 30 1 107 117 http://infolab.stanford.edu/~backrub/google.html

[B52] Culhane AC Schröder MS Sultana R GeneSigDB: a manually curated database and resource for analysis of gene expression signatures Nucleic acids research 2012 40 D1 1060 1066 10.1093/nar/gkr901 PMC3245038 22110038

[B53] Su GH Hruban RH Bansal RK Germline and Somatic Mutations of the STK11/LKB1 Peutz-Jeghers Gene in Pancreatic and Biliary Cancers The American Journal of Pathology 1999 154 6 1835 1840 10.1016/S0002-9440(10)65440-5 10362809 PMC1866632

